# A cost comparison analysis of paediatric intermediate care in a tertiary hospital and an intermediate care facility in Cape Town, South Africa

**DOI:** 10.1371/journal.pone.0214492

**Published:** 2019-04-03

**Authors:** Kristal Duncan, Edina Sinanovic

**Affiliations:** School of Public Health and Family Medicine, Health Economics Unit, University of Cape Town, Cape Town, South Africa; Tabriz University of Medical Sciences, IR Iran, ISLAMIC REPUBLIC OF IRAN

## Abstract

**Background:**

In South Africa, 600–700 new cases of paediatric cancers have been reported every year for the past 25 years, and in the year 2000, HIV/AIDS was responsible for 42,479 deaths in children under five. These children need intermediate care but research in the field is lacking, with the few costing studies conducted in South Africa reporting a range of inpatient day costs.

**Methods:**

A retrospective cost analysis for the period April 2014-March 2015 was undertaken from the provider perspective in the public sector, using a step down costing approach. Costs of paediatric intermediate care were estimated for an intermediate care facility (ICF) and a tertiary hospital in Cape Town. Costs were inflated to 2016 prices and reported in US dollars.

**Results:**

Cost per inpatient day was $713.09 at the hospital and $695.17 at the ICF for any child requiring care at these institutions. The cost for a paediatric patient who is HIV/TB co-infected was $7 130.94 and $6 951.67 at the hospital and ICF respectively, assuming an average length of stay of 10 days. For a patient with terminal brain carcinoma the cost was $19 966.63 and $19 464.69 at the hospital and ICF respectively, assuming an average length of stay of 28 days. Personnel costs accounted for 60% and 17% of the total cost at the hospital and ICF respectively. Overhead costs accounted for 12.33% at the ICF and 4.48% at the hospital.

**Conclusions:**

The drivers of cost are not uniform across settings. Providing intermediate care at an ICF could be less costly than providing this care at a hospital, however more in-depth analysis is needed. The costs presented in this study were considerably higher than those found in other studies, however, the paucity of cost data available in this area makes comparisons difficult.

## Background

In the year 2013 HIV/AIDS and tuberculosis (TB) was responsible for 12% of all deaths in children under five in the Western Cape[1]. Additionally, according to the National Cancer Registry of South Africa 600–700 new cases of paediatric cancers have been reported every year for the past 25 years and this is likely an underestimation due to poor reporting [2,3]. In light of these figures and given that the high burden of HIV and TB often results in a crowding out effect of other diseases in acute hospitals, there is a need for facilities which care for children with HIV and TB, and other life-limiting and life-threatening diseases [4].

A point prevalence study conducted in the Western Cape province in 2009 indicated that 20% of patients who had exceeded the expected length of stay at tertiary hospitals should have been discharged to an intermediate care facility (intermediate care encompasses palliative, sub-acute and respite care) [5]. Prompted by these findings the Western Cape government released a document in 2012, detailing a policy on intermediate care [5]. However to date no cost analysis studies have been performed in South Africa to facilitate the budget planning required for this type of care.

Intermediate care can be provided via a home-based care (HBC) system or be based at facilities, preferably situated in the communities which need it. Given that HBC is not a feasible option for many households in the Western Cape due to overcrowding and poor infrastructure in homes, there is a need for facility based care [6,7]. In the Western Cape, which has a population of 6,2 million, there are only nine institutions that provide intermediate facility-based care for adults and only two which provide this care for children [8]. Both of these facilities serve communities which are severely impoverished and provide palliative, respite and sub-acute care for patients suffering from HIV/AIDS, TB, malnutrition, cerebral palsy and cancer.

A 2014 WHO report indicates that the African region accounts for almost half of the global need for palliative care, however access to this care for children in low- and middle income countries (LMICs) remains scarce and there is a paucity of research on cost estimates for paediatric intermediate care [3,9,10]. In one of the few costing studies conducted in South Africa, personnel costs were shown to account for the greatest proportion of inpatient unit costs at a district hospital, accounting for between 73–82% of total costs, and more specifically paediatric wards had personnel costs accounting for between 77–83% [11]. The generalized unit costs per inpatient day at a hospital ranged between $38.04 and $103.68 and this large range of costs is due to the wide variety of diseases being treated, which makes calculating an accurate cost difficult [11,12]. An analysis of a programme combining outreach services and in-hospital stays for providing intermediate care in Gauteng province found that personnel costs accounted for 63% of the total cost of the programme [7]. Furthermore in-hospital intermediate care had a cost of $142.00 per inpatient day, which is more than the previous study’s estimate of $103.68 and possibly indicates that intermediate care is only cheaper if provided outside of a hospital setting [7,11].

This broad range of per inpatient day costs indicates the need for costing studies in the context in which the intermediate care facilities are to be established. In this study, we estimate and compare the cost of providing the standard of care to children with life-limiting/life-threatening illnesses in a tertiary hospital and at an intermediate care facility to assist with budget allocation.

## Methods

### Study setting

A cost comparison study, undertaken from the provider perspective in the public sector, was performed using a step down costing approach. Two models located in Cape Town were selected for comparison—a tertiary children’s hospital and an intermediate care facility (ICF). The tertiary hospital is a public tertiary level hospital, and the only specialist children’s hospital in Southern Africa. The ICF is a non-profit facility and receives some funding from the district department of health, with a large reliance on private donors and has been providing sub-acute care and respite care to paediatric patients since 1965. In addition, a 10 bed paediatric palliative care unit has been in operation at the ICF since 2013. In total the home can accommodate 62 patients, with two beds in the palliative care wing for parents. The majority of patients at this intermediate care facility suffer with HIV/AIDS, TB and cancer. This ICF facility was chosen because it had a dedicated palliative care wing.

### Data collection

The cost data were retrospectively collected for the period April 2014- March 2015. The period of one year has been chosen to account for any seasonal variations which might occur, and to account for the long average length of stay of patients in ICFs.

### Data analysis

Costs were estimated as per the details given in Table 1. All the costs listed in Table 1 were then added together for each facility and divided by the total number of inpatient days at each facility, to arrive at a cost per inpatient day. Cost categories include capital (equipment, computer, furniture, buildings, and training) and recurrent (personnel, consumables, transport, laboratory costs, medical supplies, and building operating & maintenance). Drug costs were not included in this study. In accordance with the intermediate care policy, any patients admitted to an ICF have to be supplied with the drugs they require from the hospital and as such the costs for drugs will be covered by the hospital, regardless of whether the patient is at the ICF or the hospital [5]. The ICF receives donations of disposable diapers and food, which were valued according to their replacement value and included in the total costs appropriately.

**Table 1:** Intermediate care costs included in a cost comparison analysis of a hospital and an intermediate care facility in Cape Town, South Africa.

Type of cost	Categories	Costing method	Valuation method
**Recurrent costs**			
**Personnel**	Clinical staff (doctors, nurses, physiotherapists), and support staff (cleaning, cooking) and administration and management.	Percentage of time spent at each facility.	Total remuneration package costs, including professional membership fees and resettlement expenditure.
**Medical supplies**	Includes items such as syringes, bandages and other wound dressing materials.	Actual quantity consumed.	For the hospital the costs are based on provincial government tender prices. For the ICF costs are based on market price of these items from the respective supplier.
**Laboratory costs**	Diagnostic tests conducted.	Actual number of tests conducted.	Costs were obtained from the financial records at both facilities.
**Consumables**	Includes the costs of food, cleaning products, baby formula and disposable nappies.	Actual quantity consumed.	Costs were obtained from the financial records at both facilities.
**Transport**	Transport running costs- fuel and shuttle service fees.	Number of kilometres travelled.	At the hospital fuels costs and vehicle maintenance costs were obtained from financial records. At the ICF costs are based on invoices from the shuttle service provider.
**Building operating & maintenance**	Water, electricity and contracted services.	Actual quantity attributable to each facility.	Costs were obtained from the financial records at both facilities.
**Capitals costs**			
**Building costs**	The facility structures and all attached offices.	The building cost per m^2^ was set at R40000, which includes inside finishes, and this value was supplied as a standard by the provincial department of health.	The area of the ICF was calculated using Google Earth Pro [16]. The area for the hospital was supplied by the facility.
**Equipment**	Medical and non-medical equipment, furniture, staff uniforms and linen		The current useful life span of each asset was determined and the current replacement value of the asset calculated.
**Training**	Expenditure on training of staff at each facility.	Actual expenditure on staff training, at each facility.	Costs were obtained from the financial records at both facilities.

The cost per inpatient day for each facility was estimated, and then inflated to the 2016 prices using an average annual inflation rate of 6.1% [13]. To allow for the differential timing of capital items, these costs were annuitized using a discount rate of 3% in accordance with international standards [14]. The costs are reported here in US dollars (the average exchange rate for 2016 of $1 = R14.87 was applied) [15].

Utilization rates at both facilities were comparable at approximately 83%. The inpatient days for the ICF were calculated using the monthly bed occupancy rate provided by the facility for the study period. The number of inpatient days at the hospital for this same period were provided by the hospital’s financial management unit.

### Average length of stay for proxy cases

Due to the wide array of diseases requiring intermediate care and the lack of disease-specific ALOS information, two diseases were used as proxies for the average length of stay, namely patients with HIV/AIDS with a co-infection of tuberculosis (TB) and patients with terminal brain carcinoma. These are among the most common diseases being treated at the ICF, and are among the top four diseases requiring intermediate care, according to the Western Cape Department of Health [5,11,12]. The only value that the hospital could provide was a general ALOS for the entire hospital, which was 3.9 days for the period of analysis. At the ICF there was only limited information available, with the length of stay for patients with HIV/TB co-infection ranged from 6–644 days and for terminal brain carcinoma the length of stay ranged from 407–604. Given this extremely varied length of stay, the ALOS was determined using what was available in the literature. International literature indicates an ALOS of 8–11 inpatient days for children receiving care for HIV-related admissions, with a South African study finding a mean of 12.7 inpatient days [17–19]. Therefore an ALOS of 10 days was used for the proxy case of a child who is HIV positive and co-infected with TB. Only one study has been published on the ALOS for terminal brain carcinoma in children, indicating an ALOS was 28.9 days [20]. Given this, a period of 28 days was used for the proxy case of a child who has a terminal brain carcinoma.

### Sensitivity analysis

A one way sensitivity analysis was performed, testing one parameter at a time. The first parameter tested was the ALOS due to the diversity of ALOS in the literature. The lowest ALOS was 3.9 days (general ALOS at the hospital according to records), while the highest was 498 days, as determined through a review of patient files at the ICF. Thus for scenario one the lower bound value of 3.9 days and the upper bound value of 498 days was used for ALOS. The second parameter tested was the discount rate. A discount rate of 6% was used to test the assumption of using a discount rate of 3%.

### Ethics

Ethical approval for this study was obtained from the University of Cape Town’s Human Research Ethics Committee (HREC REF 249/2015). In addition, institutional approval was obtained from the Board of Trustees at the ICF and from the manager of medical services at the hospital.

## Results

### Per inpatient day costs

Cost per inpatient day was $713.09 at the hospital and $695.17 at the ICF. The difference in the cost per inpatient day between these two facilities was minimal at $17.93 (Table 2). The cost for a paediatric patient who is HIV/TB co-infected was $7,130.94 and $6,951.67 at the hospital and ICF respectively, assuming an average length of stay (ALOS) of 10 days. For a patient who has a terminal brain carcinoma the cost was $19,966.63 and $19,464.69 at the hospital and ICF, respectively, assuming an ALOS of 28 days.

**Table 2:** Summary of unit costs for the hospital and the intermediate care facility in 2016 US $.

	Hospital		Intermediate care facility		
**Type of cost**	**Cost per inpatient day ($)**	**Percentage of total cost**		**Cost per inpatient day ($)**	**Percentage of total cost**
**Capital costs**	**141.46**	**19.8%**		**415.26**	**59.7%**
Equipment, computer, furniture	33.49	4.7%		32.01	4.6%
Building costs	107.50	15.1%		372.77	53.6%
Staff Training	0.47	0.1%		10.47	1.5%
**Recurrent costs**	**571.63**	**80.2%**		**279.93**	**40.3%**
Personnel	424.33	59.5%		108.64	15.6%
Consumables	43.89	6.2%		45.85	6.6%
Transport	0.06	0.0%		23.06	3.3%
Laboratory costs	21.39	3.0%		0.34	0.0%
Medical supplies	38.29	5.4%		0.43	0.1%
Building operating & maintenance	43.66	6.1%		101.61	14.6%
**TOTAL**	**713.09**			**695.19**	

### Largest cost drivers of overall cost

**Fig 1:**
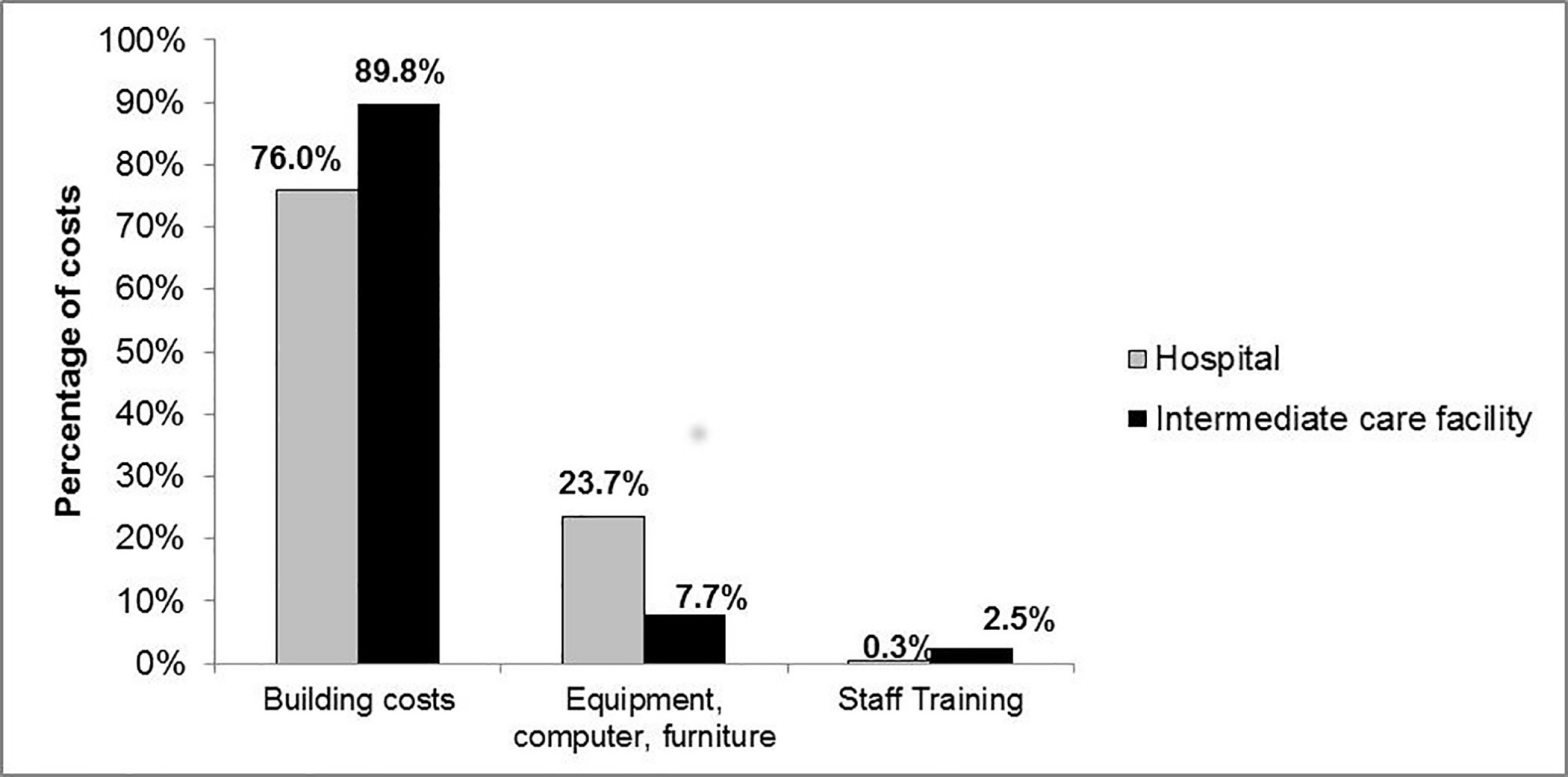
Breakdown of capital costs for the hospital and the intermediate care facility.

Analysis of each of the cost types highlights the different cost drivers at the two facilities. At the hospital, recurrent costs account for around 80% of total costs, while at the ICF capital costs are the biggest cost drivers, accounting for nearly 60% of total costs (Table 2). A detailed analysis of capital costs indicates that equipment costs account for nearly a quarter of the total capital costs at the hospital, compared to the ICF, where less than a tenth of the capital cost is attributable to equipment (Fig 1). For both facilities building costs account for the largest proportion of capital costs, 76% and 90% at the hospital and the ICF respectively (Fig 1).

**Fig 2:**
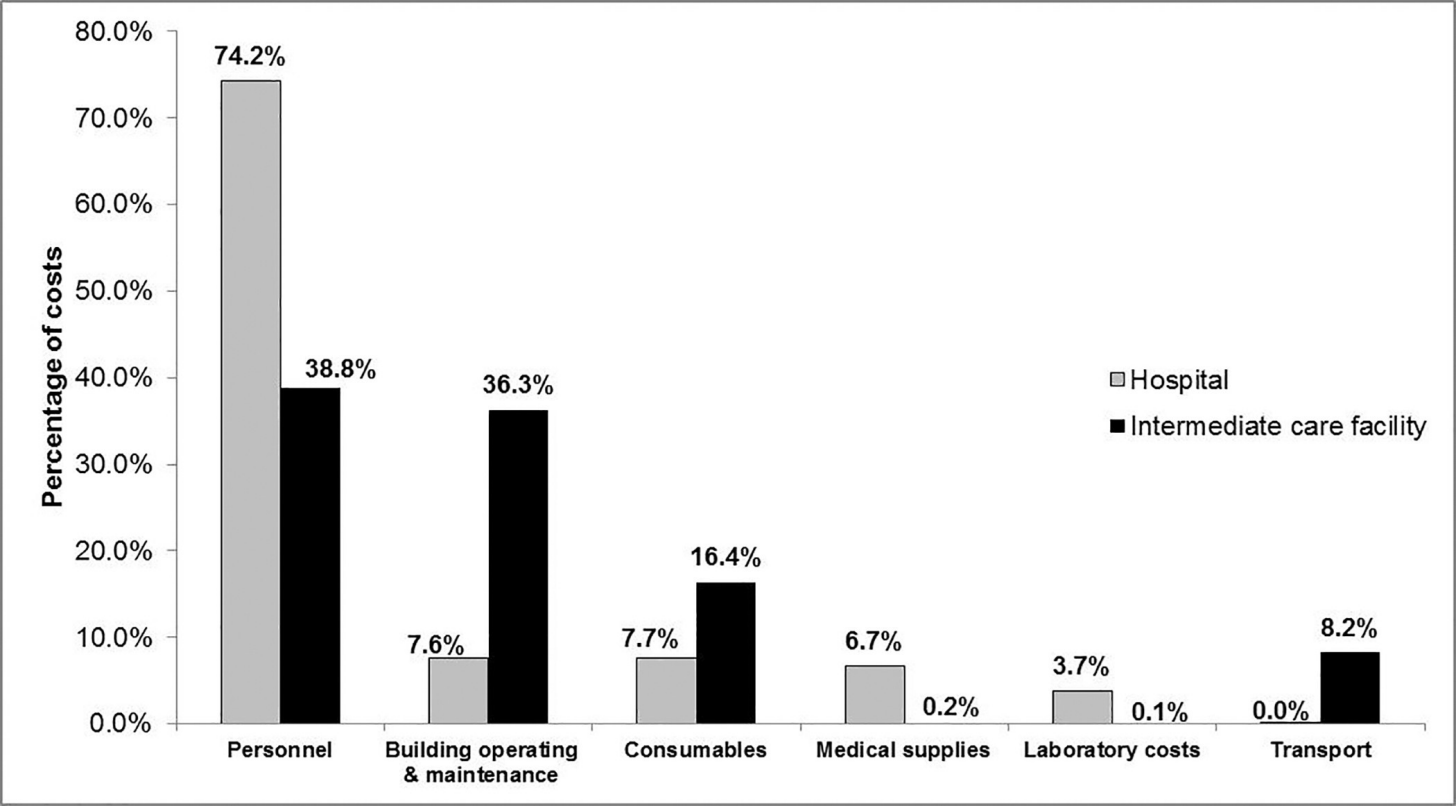
Breakdown of recurrent costs for the hospital and the intermediate care facility.

Personnel costs are the largest contributors to recurrent costs at both facilities, accounting for nearly 75% of all recurrent costs at the hospital, compared to only 39% at the ICF (Fig 2). Building operating and maintenance costs are also a large contributor at the ICF (36.3%), but are only a marginal fraction of recurrent costs at the hospital (7.6%) (Fig 2).

**Fig 3:**
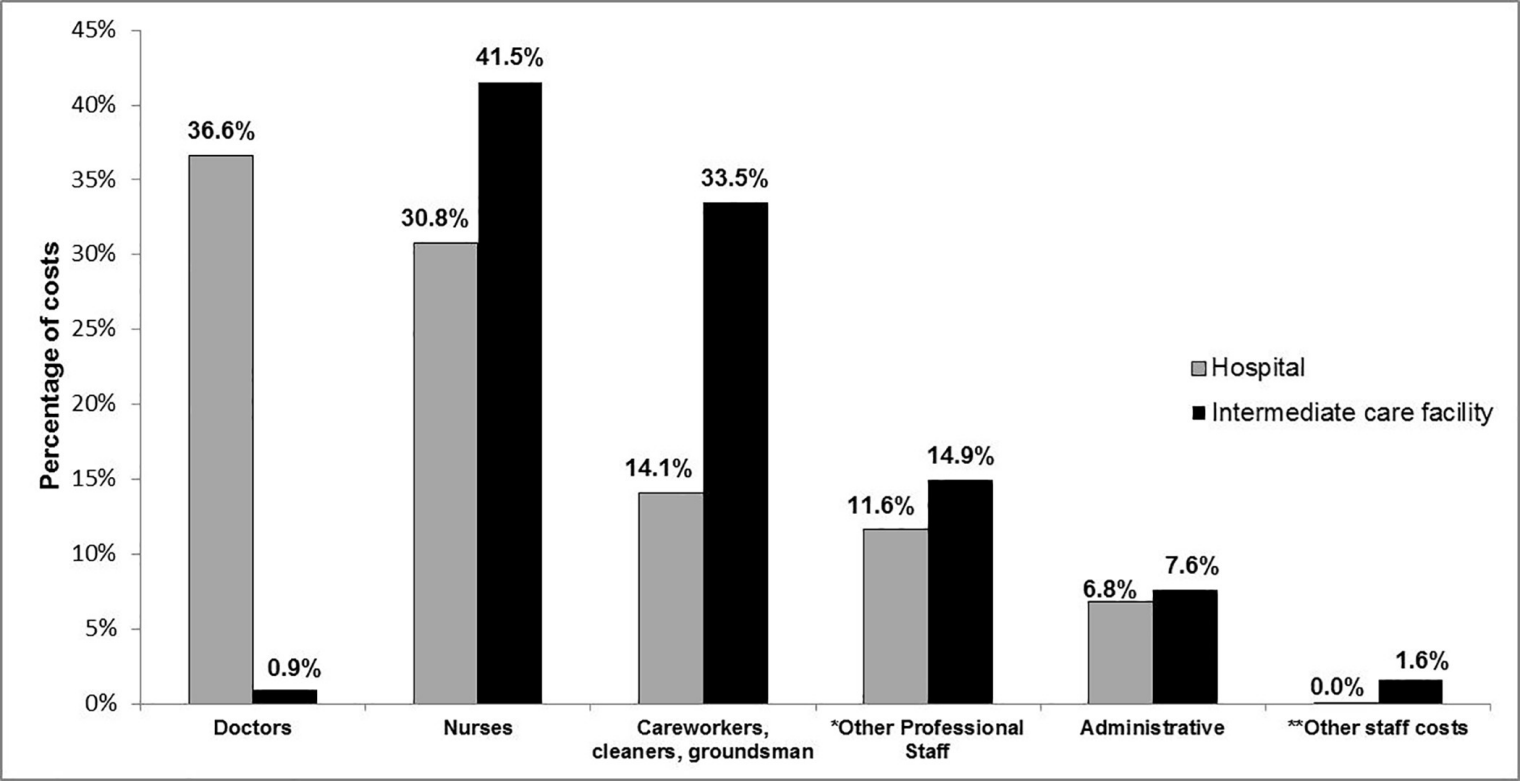
In-depth analysis of personnel costs for the hospital and the intermediate care facility. *Other Professional staff includes dieticians, physiotherapists and laboratory technicians **Other staff costs include labour settlement, resettlement fees and membership fees.

Given the large contribution of personnel costs to the total recurrent costs, a more in-depth analysis was performed. Administrative staff, nurses and other professional staff (dieticians, physiotherapists and laboratory technicians) have comparable contributions to the total personnel costs at each facility. Noticeable differences can be seen for doctors, who contribute 37% of personnel costs at the hospital compared to only 1% at the ICF (Fig 3).

### Sensitivity analysis

Under all of the scenarios the original outcome that per inpatient day costs at the ICF are cheaper than at the hospital, holds true (Table 3). Under the first scenario, the lower bound ALOS of 3.9 days results in a saving of $4,240.51 and $4,349.87 per inpatient day at the ICF and hospital respectively. While the upper bound ALOS of 498 days results in an increase in unit cost of $326,728.68 and $335,154.17 at the ICF and hospital respectively. A discount rate of 6% instead of 3% results in the unit cost being $71.70 higher at the hospital than the ICF; a difference which is 4 times greater than that calculated in the baseline scenario.

**Table 3:** Effect on unit cost by differing assumptions of the baseline case in 2016 US $.

		ICF		Tertiary hospital
**Baseline**		Unit cost ($)		Unit cost ($)
	Total per inpatient day cost	695.17		713.09
	Cost for TB/HIV co-infected patient	6,951.67		7,130.94
	Cost for terminal brain carcinoma patient	19,464.69		19,966.63
**Scenario 1**	Total per inpatient day cost	695.17		713.09
	Cost for ALOS of 498 days	346,193.37		355,120.80
**Scenario 2**	Total per inpatient day cost	1,304.64		1,394.26
	Cost for TB/HIV co-infected patient	13,046.42		13,942.65
	Cost for terminal brain carcinoma patient	36,529.97		39,039.41

## Discussion

The total costs identified in this study are considerably higher than the costs in other South African hospital costing analyses. Previous studies in district hospitals put the cost per inpatient day between $37.23 and $212.09, for various programmes within a district hospital setting [7,11]. While a study in West Africa puts the total cost of care for an HIV-infected child at between $42.53 and $123.39[21]. The unit cost reported here is almost four times the highest inpatient costs cited in South Africa previously, however these estimates are for a district hospital not a tertiary hospital [7,11]. Another possible explanation for this difference could be the broad scope of the current study which is to assess care provided to patients with a range of diseases, whilst previous studies have focussed on only one department or programme, and not an entire hospital. The proportion of costs attributed to the various cost centres within a hospital setting is in line with previous literature, where in district hospitals in South Africa personnel costs were found to account for between 63% - 82% of the total costs [7,11]. It is difficult to make comparisons with the findings here for intermediate care facilities, as no such studies have been done in South Africa or LMICs previously, and those that do exist are for specific diseases such as HIV[21]. However overhead costs are a large driver of cost at the ICF, which is contrary to what would be expected, given that this facility is far smaller than the hospital. This finding highlights the need for further investigation into these costs and possible ways in which they could be reduced. If the ICF had been fully funded by the Western Cape Department of Health for 2016, the cost would have been 0.02% of the total budget for the province [22]. Given that this is a large percentage of the total provincial budget for a 62 bed facility, more detailed economic evaluations are needed to determine whether this type of care in its current format is cost-effective.

It should be noted that since conclusion of this research the palliative care wing at the ICF was shut down due to a lack of funding and the patients who were in the wing were integrated into the general ward until an alternative arrangement could be made. The issue of providing intermediate care is therefore a complex one, compounded by the fact that this type of care is provided across a range of diseases. However, despite the fact that the findings of this study indicate that cost of care at a ICF is not much cheaper than at a hospital, given the added benefit which families obtain from being supported by health care providers who are trained in intermediate care specifically, it is imperative that more research in this area is conducted in order to equip policy makers with the necessary evidence base with which to implement policies effectively [23–27].

Prior to the commencement of the study it was clear that facility management assumed that ICFs are less costly largely due to the fact that they are staffed by nurses, while doctors only work on a part-time basis, as per the guidelines for intermediate care in the province[5]. The findings here indicate that personnel costs are indeed far lower in these facilities than in a tertiary hospital, with doctors accounting for a very small amount of personnel costs overall at the ICF. Another area which is less costly is evident upon closer analysis of the capital costs. Equipment costs at the hospital account for just under a quarter of the total capital costs, compared to only 7% at the ICF and therefore in certain cost categories the ICF is the less costly option. However, policies should give guidance on possible ways to reduce overhead costs as these were found to be a large driver of costs at the ICF, and these costs should be minimized if these facilities are to become truly cost-saving. Given that the building costs both in terms of capital cost and recurrent costs (in the form of maintenance) are large cost drivers, it may be less costly if the ICF was located on hospital property. This is one way in which the cost-savings seen in personnel and equipment costs could be maximized.

Lastly, while the current study illustrates the high cost of care from a provider perspective for children with life-threatening and life-limiting diseases, it did not include costs from the patient’s perspective. While it is probable that some cost-sharing does occur even in the public sector, given that the population served by the ICF has a relatively low socio-economic status, it is reasonable to omit the patient’s perspective for this current study. However future work should address this issue. Nonetheless other research suggests that these costs, which include transport costs and lost wages, are high and future research should aim to quantify these costs in LMICs [28]. Given the challenges with assessing the quality of care and outcomes associated with care provided at the two facilities under comparison here, a more in-depth, full cost-effectiveness assessment of intermediate care provided at an ICF compared to a district or tertiary hospital is needed. However this study is a first step towards addressing the numerous calls for increased research activity in the field of paediatric intermediate care in Africa [29]. Furthermore while the difficulty with estimating health outcomes in children is well-documented, future studies should investigate the cost-effectiveness of providing intermediate care at a ICF, versus providing the same care at a hospital [29,30]. The next step for research in this area of paediatric palliative care in South Africa should attempt to understand differences in the ALOS and the outcomes of the care received for the wide variety of patients who receive intermediate care at both ICFs and tertiary hospitals. One way in which this could be done is to prospectively document the costs and outcomes associated with providing care to a small sample of patient who require intermediate care in the Western Cape.

## Conclusions

Cost comparison studies in the area of paediatric intermediate care are lacking generally and even more so in South Africa and other LMICs. Despite the limitations of this study due to a lack of available data in this field, the findings may suggest that this type of care is less costly if provided at an ICF than at a hospital, however there is the possibility for further cost reductions, especially with regards to overhead costs. In conclusion, more research in this field is needed if ministries of health are to provide interventions which are cost-effective and meet patient needs, and devise policies which are feasible given budgetary constraints in the health sector.

## Supporting information

S1 File Raw cost data for the ICF and tertiary hospital. Raw unit costs for each institution being analysed, broken down by capital and recurrent costs. Costs are presented in ZAR and USD and the relative contribution of each type of cost to overall costs is calculated.
(XLSX)
